# Virtual Faculty and Peer Mentoring to Promote Social Belonging among Minoritized Physical Therapist and Nursing Students

**DOI:** 10.3390/healthcare10030416

**Published:** 2022-02-23

**Authors:** Keshrie Naidoo, Laura Plummer, Martha McKean, Amanda Mack, Garrett Kelley Bowdle, Margaret Anne Mullins, Shweta Gore

**Affiliations:** Department of Physical Therapy, MGH Institute of Health Professions, Boston, MA 02129, USA; lplummer@mghihp.edu (L.P.); mmckean@mghihp.edu (M.M.); amack@mghihp.edu (A.M.); gbowdle@mgh.harvard.edu (G.K.B.); mmullins4@partners.org (M.A.M.); sgore@mghihp.edu (S.G.)

**Keywords:** minoritized students, health sciences, virtual mentoring, social isolation, pandemic

## Abstract

Minoritized health sciences students report experiencing social isolation and discrimination, and cite the lack of faculty representation as barriers to their success. While virtual mentoring can increase sense of belonging and connectedness, these effects have not been examined in minoritized health sciences students. The purpose of this study was to investigate whether virtual mentoring from faculty and peers could decrease social isolation and promote social belonging among minoritized first-year physical therapy and nursing students. Using a mixed methods explanatory sequential design, racial and ethnic minority physical therapy and nursing students (*n* = 8) received virtual mentoring and attended virtual networking events while students from across the health profession programs served as a comparison group (*n* = 16). While virtual mentoring relationships took longer to establish, there was an increase in satisfaction with mentoring for the intervention group compared with no improvement for the comparison group who received traditional academic advising. Qualitative data analysis revealed that mentors served as role models who had overcome barriers and persevered, decreasing feelings of isolation, and bolstering mentee confidence. A virtual multiple-mentor model can decrease isolation and promote social belonging for minoritized students and offer support for students even after the pandemic.

## 1. Introduction

The critical need for a racially and ethnically diverse healthcare workforce to meet population needs is evident [1]. However, despite the growing diversity of the USA population [2], only 10 percent of healthcare professionals practicing in the USA are from minoritized backgrounds [3]. Even nursing, regarded as one of the most racially diverse health occupations in the USA [4], is 75.4% non-Hispanic White [5]. The physical therapy (PT) workforce is 84.3% White [6], and racial and ethnic minority (REM) students in accredited Doctor of Physical Therapy (DPT) programs make up only 23.3% of graduates [7]. Furthermore, REM students have increased academic difficulty while enrolled in DPT [8,9,10] and nursing (NS) programs [11,12], and underperform on national licensing tests compared to their White counterparts [13]. NS and PT students report social isolation, discrimination, stereotyping, and a lack of representation in academic and clinical faculty as factors which contribute to academic difficulty [8,14,15,16,17].

Mentoring in graduate health sciences programs can decrease social isolation [18] and increase the recruitment and retention of REM students [19,20,21,22]. REM students report that mentors help introduce them to the unwritten norms and rules of the profession, role-model effective professional behavior, and improve their social capital by introducing them to a professional network [23]. However, approaching faculty mentors can be a barrier for REM students, and senior students, who can provide information and support, are a helpful conduit between faculty and first-year students [24]. While virtual mentoring can increase students’ sense of belonging, and foster community and connectedness [25,26,27,28], these effects have not been examined in REM health sciences students. Therefore, the purpose of this study was to investigate whether virtual networked mentoring from faculty and REM peers could decrease social isolation and promote social belonging among REM students enrolled in the first year of a graduate health sciences program.

### Theoretical Frameworks

The first theoretical model which situates this study is Tinto’s theory of university departure [29], which highlights academic and social integration as vital to student retention. Social integration factors include relationships with peers and informal interactions with faculty [29]. The second theoretical model, the racial/cultural identity development model [30], offers insight into how REM students may acclimate to the higher education environment at primarily white institutions (PWIs). In the initial stages of this model, REM students often conform to the dominant culture; however, over time, the student comes to appreciate the positive aspects of their own culture. Eventually, the REM student values aspects of both their own culture and those of the dominant culture [30]. When exploring the need for mentorship in higher education, this model has implications for the racial congruence of mentoring teams and suggests the consideration of mentoring that breaks from the traditional 1:1 model.

## 2. Materials and Methods

### 2.1. Study Design and Context

**Figure 1:**
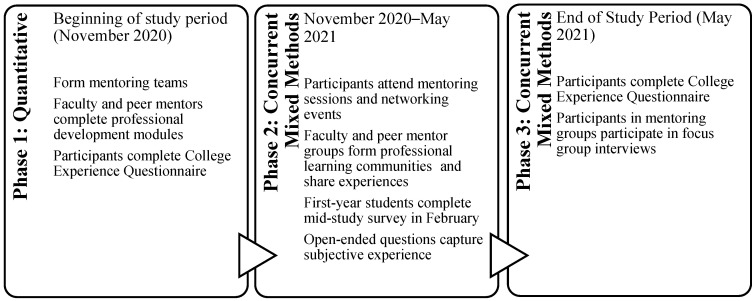
Data collection using the mixed methods (explanatory sequential) design.

This study leveraged a mixed methods explanatory sequential design [31] (see Figure 1). The context was a health sciences graduate school in the northeast region of the USA with PT, NS, genetic counseling (GC), occupational therapy (OT), physician assistant studies (PA), and communication sciences and disorders (CSD) programs. At the time of the study, the school included 1756 full-time students and 118 full-time faculty members. The study duration was six months, beginning in November 2020.

### 2.2. Participants

First-year students from GC, OT, PT, PA, CSD, and NS programs (N = 336) were recruited via purposeful sampling into an intervention group that received faculty and peer mentoring or a comparison (usual care) group (see Table 1). Inclusion criteria for the intervention group included being enrolled in the first year of a graduate program and self-identifying as REM on application materials (*n* = 108). Students were excluded from the intervention group if they did not identify as REM, declined to share race upon application, or were already enrolled in a mentoring intervention. All first-year graduate students were eligible to participate in the comparison group. First-year students self-selected into the intervention or the comparison group. Second-year REM graduate students were recruited using snowball sampling to serve as peer mentors and were offered a $200 stipend. To eliminate the effect of coercion between researchers, some of whom were faculty at the institution under study, and student subjects, program staff distributed recruitment materials via email. Faculty were purposively recruited from the same program as the first-year students in the intervention groups to participate as mentors.

### 2.3. Intervention

The intervention was based on a pilot study [18] and included a combination of: (1) faculty-directed mentoring; (2) peer mentoring from second-year REM students; and (3) participation in networking events. Mentors completed asynchronous online training modules and formed separate professional learning communities (PLCs) through online discussion boards and virtual meetings with the study team. Peer mentor professional development topics included: (a) an introduction to the theoretical frameworks which situated the study; (b) the power of peer mentoring; (c) the relationship between social belonging and academic outcomes; (d) facilitating difficult conversations; and (e) the three pillars of mentorship [32]. The three pillars of mentorship include: (a) knowledge of mentoring on the run; (b) creating a community of mentors; and (c) facilitating a culture of mentoring. Mentoring on the run describes a shift from thinking about mentoring as a formal interaction to infusing mentoring into daily interactions [32].

Faculty were introduced to the five-tier mentoring model [33] that included (a) commitment to the mentoring process, (b) establishing mentoring venues, (c) serving as a role model, (d) employing successful tools, and (e) monitoring mentee’s progress. Strategies for mentoring REM graduate students [34] included helping mentees expand their contacts, sharing personal stories, using humor, responsiveness (which conveys accessibility), and validation balanced with constructive feedback. Study staff met with faculty and peer mentor groups at the beginning of the study and mid-study to review and refine mentoring strategies.

### 2.4. Mentoring Teams

The team included a first-year mentee, a matched faculty mentor, and a REM peer mentor from the same health sciences discipline. Given the small sample of REM students, it was unlikely that racial concordance between mentees and peer mentors would be achieved. Instead, it was anticipated that mentees and peer mentors would leverage a shared experience as health sciences students from a minoritized background enrolled at a PWI. As only 10% of faculty at the institution identified as REM, researchers did not consider race when recruiting and matching faculty mentors. Teams were asked to meet a minimum of six times (either as faculty–mentee or peer mentor–mentee dyads or a group) over the six-month study period (approximately once a month) with recommendations to guide each interaction. Due to social distancing measures, mentees met with faculty and peer mentors virtually via Zoom. The first session focused on establishing a social connection, highlighting shared experiences and motivations to pursue a career in the health professions. Subsequent sessions were dedicated to establishing the mentoring relationship, discussing the culture of the profession, mentee goals for the future, barriers to success, and debriefing after networking events. No recommendations were made about the length of each mentoring session. Peer mentors also met with faculty mentors in 1:1 meetings to advocate for mentees and guide faculty towards meeting REM students’ needs.

### 2.5. Networking Events

Tinto’s model highlights that increasing student, peer, and faculty contact is vital to increasing social and academic integration of REM students in higher education [29]. Therefore, in addition to mentoring meetings, mentees as well as their faculty and peer mentors attended three 90-min virtual networking events hosted via Zoom which introduced mentees to an interprofessional group of REM leaders in the health sciences field. The first event included a panel presentation on service-learning, while the second included an interprofessional REM faculty panel who shared the barriers and facilitators to their success. In the final event, faculty mentors invited and interviewed their own mentors, who had helped them achieve career goals, to highlight strategies for finding and working with a mentor throughout a professional career. During all networking events, mentees had the opportunity to ask questions of the panelists and debriefed with their mentors about the event.

### 2.6. Comparison Group

It is possible that social isolation could decrease over the course of the two-semester intervention regardless of the intervention. For this reason, a comparison group of first-year students was recruited. Students in the comparison group received “usual care” which included meeting with academic advisors. Additionally, some students were assigned a second-year peer buddy by their program. Peer buddies received no formal training for this role. Students in the comparison group did not attend networking events.

### 2.7. Measures

We employed the College Experience Questionnaire (CEQ) and Mentoring Survey. The CEQ [35,36] is a 21-item questionnaire that includes three subscales: university environment, connectedness, and alienation, and is designed to assess graduate students’ sense of belonging and connectedness with an acceptable internal consistency (Cronbach’s α 0.78) [35]. Students in the intervention and comparison groups completed the CEQ electronically at the beginning (November 2020) and end of the study (May 2021). Participants completed mid- and end of study surveys developed during the pilot study [18] to track the dosage of the intervention received. The surveys were designed to collect data on the number of mentoring sessions received and number of networking events attended. There were also open text fields for student comments about the mentoring intervention.

Focus groups with all eight participants from the intervention group and were held at the end of the study. Two focus groups included 3 participants and 1 focus group interview included 2 participants. One participant, unable to attend the focus groups, participated in a 1:1 interview. The structured interview guide [18,36] explored mentoring and social belonging (see Appendix A). Subjects were asked not to reveal identifying information about themselves and others in the group and asked that all information shared during the focus group be kept confidential.

### 2.8. Analysis

Descriptive statistical analysis followed quantitative data collection. All quantitative data analysis was performed using IBM SPSS version 25.0 (IBM Corp, Armonk, New York, NY, USA). Open coding was used to analyze focus group interview data, researcher field notes, and open-ended survey responses using NVivo qualitative software (QSR International Pty Ltd., Doncaster, Australia, 2020). After the second cycle coding, codes were collapsed into themes [37].

### 2.9. Trustworthiness

To increase credibility, researchers leveraged data triangulation and an audit trail including the use of multiple data sources: surveys, focus group interview transcripts, and field notes [38]. As the researchers adopted a constructivist paradigm, during thematic data analysis, thick, rich descriptions were used to work towards credibility in this analysis [39]. All research materials were kept in a central location to produce an audit trail and allow for the study process to be replicated.

## 3. Results

Eight first-year students (four DPT and four NS students) consented into the intervention group. Mentees self-identified as African-American/Black (*n* = 4), Asian/Pacific Islander (*n* = 2), or Hispanic (*n* = 2). Sixteen students from across the health professions programs at the institution consented into a comparison group (See Table 1). Eight faculty mentors included one associate professor, four assistant professors, and three instructors. Two faculty mentors belonged to the REM groups. Seven peer mentors included four DPT and three NS students, one of whom served as mentor to two NS students. All DPT peer mentors had been first-year mentees in the previously published pilot study [18]. Participants in the intervention group (mentees) met with their faculty mentors an average of five times, while participants in the comparison group met with faculty advisors twice over the same period (standard practice at the institution under study). Mentees also met with their peer mentors five times over the study period compared with participants in the comparison group who did not meet with the peer buddies who may have been assigned by their program.

Mentee perception of faculty involvement in student well-being during the mentoring meetings increased towards the end of the study (Table 2). At the beginning of the program, seven participants (87.5%) agreed that the faculty mentors reviewed their challenges and concerns in the meetings. By the end of the program, all mentees agreed with this statement. In the comparison group, 11 participants (68.6%) agreed that faculty addressed their challenges in the program, and 10 participants (62.5%) agreed that faculty addressed their concerns by the end of the study period.

Research Question 1: To what extent does participation in a virtual multiple-mentor model decrease feelings of isolation for first-year REM health sciences students?

CEQ Subscale—University Alienation: The university alienation sub-scale explored social and racial isolation and feelings of inadequacy while enrolled in graduate school. The intervention group did demonstrate a decrease in alienation scores at the end of study. At the beginning of the study period, the intervention group neither agreed nor disagreed with the statement “I feel racially isolated” but by the end of the study period, the intervention group disagreed with this same statement. Of note is that the comparison group strongly disagreed with this statement at both time periods (Table 3). Analysis of the focus group data supported findings of social isolation at the beginning of graduate school but also highlighted a decrease in social isolation through mentoring.

Theme: Closing Social Distances. Participants, who began graduate school during the pandemic, described difficulty identifying a typical graduate school experience and missed the social aspect of their experience. Without an in-person orientation and few campus visits, participants felt isolated from their classmates. While participants were able to participate in clinical education, at clinical facilities participants noted that the only other minorities were maintenance staff, leading to further isolation. Participants began to wonder if they belonged in graduate school, “I was questioning if I was even smart enough to be here” [Focus Group (FG)4, Participant (P)2]. Participants were also distanced from their chosen professions, describing healthcare as “dynastic.” One participant described, “I knew I wanted to be a nurse, but I didn’t really know what that meant” and questioned their career trajectory, “It made me question whether there would be support or if existing in this field would be a constant fight for my whole career” (FG 2, P1). Mentors were able to provide valuable insight into school and the profession and guide mentees towards navigating challenges:

*“Both [faculty and peer] mentors helped me because I was getting stressed out about possibly wanting to switch my specialty, and it was something that had been on my mind for a long time… I did end up changing my specialty, because of their advice, so I think both of them helped me in terms of my future and just in general.”*

(FG 4, P2)

In addition to the pandemic, students also started graduate school during a tumultuous socio-political climate in the USA. Participants expressed frustration with the long fight for racial justice and questioned whether enough was being done. Students described the challenge of trying to focus on school while there were widespread protests going on and the associated anxiety:

*“I’m not saying that I am just my race, I am just my culture, but like you can’t ignore it, and you can’t ignore what is going on outside in the world. We’re not in our own little bubble, I wish we were sometimes.”*

(FG4, P2)

Participants were also experiencing racism in their immediate contexts. One participant described this incident while working as a nursing assistant in a psychiatric hospital:

*“There was a patient who I had a really hard time with, who was really inappropriate, used the “n-word” all the time, and it was really draining to keep interacting with this person on a constant basis. When I had brought it up to other people, they tried to always give me advice on how to steer clear from it or kind of dismiss it and say, “It’s this person’s illness.” … which is true. But I think that those are all answers which centered the patient.”*

(FG 2, P1)

While the participant understood the patient’s cognitive limitations, they left the experience feeling unsupported until they shared their experience with their faculty and peer mentor. The mentoring team offered a safe space where they felt heard and validated:

*“My peer mentor shared some of his own experiences working in health care and interacting with patients who were being racist. It felt really personally validating and made me feel like it’s okay, not everything is always about the patient. When I am removed from the situation, when I am at home, I can make it about myself.”*

(FG 1, P2)

Ultimately, mentors served as role models who had overcome barriers and persevered, reassuring participants, decreasing feelings of isolation, and bolstering confidence:

*“[The mentors] have personal experiences that make them who they are, and overcoming some challenges, and making me feel like I am not alone and I can do it too. They are smart, intelligent people, and resilient. Learning from their personal experiences I felt a little more confident too, that I can do this.”*

(FG 1, P2)

Research Question 2: To what extent does participation in a virtual multiple mentor model promote social belonging for first-year REM health sciences students?

University Connectedness and Environment: These subscales focused on a sense of entitlement, belonging, and the ability to freely express opinions at school. There was a trend towards decline in connectedness scores among the intervention group for all items except for one. Scores improved at the end of study for “I feel fully entitled to all of the resources available on campus” (see Table 3). In terms of the university environment, participants in both the intervention and comparison groups disagreed that there were sufficient minority faculty at the institution. In contrast to CEQ findings, qualitative data supported an increased sense of connection to mentors and access to a support network.

Theme: Shared Learning Spaces. Participants described that initially graduate school was “the great unknown” and shared that their mentors provided resources and connected them to a network: “So as a first-year student, and first-generation also, I feel like I don’t know what I don’t know… building the network with the peer mentor and faculty mentor, I felt that I got to know more resources” (FG 1, P2). When asked who they would turn to with questions if they did not have mentors, one participant remarked, “Honestly I’m not sure who I would go to” (FG 3, P2). Another participant shared that even when their mentors did not have a solution, the mentors were able to refer them to someone else because of better connections, “I think without that structured relationship I probably wouldn’t have answered those questions and probably would have been a lot less prepared” (FG 2, P1).

Participants appreciated that achieving racial concordance in mentoring was challenging but still learned from the experience:

*“We’re all not going to be able to have mentors of people exactly from your background. It’s nice when it happens because they share that perspective with you, but as we’re going to be moving through life, having to build relationships with people of other backgrounds, having to be comfortable in White spaces. I think that was still helpful.”*

(FG 4, P1)

While some participants described a connection to their mentor and not necessarily the school, for most, the mentorship program made them feel invested in, and ultimately created a connection to the school:

*“I definitely did not feel any sort of attachment to school at all prior just because of everything being remote and not being able to connect with any other students and I think the mentorship program definitely helped me feel some attachment to school.”*

(FG 2, P1)

As two of the faculty mentors and all the peer mentors identified as being of racial/ethnic minority, participants described being greatly concerned adding to the invisible labor of minority faculty and students:

*“This was a wonderful experience for me, and this experience, for other students, to make it more sustainable, I worry about faculty of color and students of color, their invisible labor or the time and energy. This is energy consuming too, listening to what I have to go through, the challenges.”*

(FG 1, P2)

While participants reported that virtual relationships took longer to establish, they began to appreciate the bidirectional nature of the mentoring. Participants valued the role that their interaction with a group of mostly White faculty mentors played in promoting racial understanding and enhancing the educational experience for other students of color:

*“I think it’s important for faculty of any background to take part in these types of studies because it allows them to meet and understand and start to just talk to students from different backgrounds and start building those relationships that might not be built in the classroom.”*

(FG 4, P1)

## 4. Discussion

It is challenging to differentiate REM students’ feelings of isolation due to the pandemic or from being a minoritized student at a PWI, an environment which may be incongruent with their culture and values [14]. The pandemic and the associated social distancing measures likely exacerbated existing challenges for REM students in higher education, highlighting the need for non-traditional virtual mentoring models which leverage support from minoritized peers. Virtual mentoring provides an efficient and available form of support for students [40,41], but this effect had not been studied in REM health sciences students until now.

Participants in the intervention and comparison groups described feeling socially isolated at the beginning of the study period; however, REM participants in the intervention group also faced racism and discrimination. Findings of bias and discrimination towards REM students in science, technology, engineering, and medicine (STEM) are not uncommon [8,14,17,42], and can lead REM students to question their abilities within STEM fields [43]. In this study, participants questioned whether they belonged in their health sciences field, but felt validated by mentors who role-modeled perseverance. Peer mentors served as relatable mentors who role-modeled success, highlighting that quality relationships with fellow students can foster social integration and contribute to academic persistence and success [44,45,46]. Qualitative data revealed that participants felt less isolated after interacting with mentors and grew more confident in their ability to succeed in their chosen professions. These findings replicate those of the pilot study, which was implemented in person [18], again highlighting the value of virtual mentoring.

Few mentors receive training on how best to meet the unique mentoring needs of REM students [21] who have additional challenges to overcome, such as discrimination, decreased confidence, and social isolation [18]. In this program, mentors completed mentor training and met with study stuff to refine mentoring strategies. While participants reported that the virtual mentoring relationships took longer to establish, which is consistent with existing literature [40,41], mentee perception of mentoring improved over the course of the study.

Surprisingly, CEQ university connectedness scores decreased over the course of the study, potentially due to the pandemic which limited participants access to campus and contact with peers and faculty. Another potential explanation is that while participants described feeling connected to their mentors, this did not necessarily translate to a sense of connectedness to the institution. The one item that increased was entitlement to resources on campus, as participants were exposed to a larger network by their mentors, supporting findings about the benefits of faculty and peer mentors.

### Limitations

This study was conducted during the pandemic, which is a unique context for studying mentoring and a potentially confounding factor. While participants were recruited from six professional programs, only eight REM students from DPT and NS programs (out of an eligible 108 students) consented into the intervention group. A confounding variable is that the intervention and comparison groups differed at baseline in terms of racial isolation offering some insight for motivation to participate in the mentoring portion of the study. The reason for the low enrollment is unclear, as students who did not consent into the study were not interviewed. It is possible that the advising structure at the institution under study (which includes academic advisors and peer buddies in some programs) may provide students with sufficient support. Ultimately, a small group of participants from two programs at one institution limits the generalizability of findings. Additionally, the CEQ is designed to assess graduate students’ sense of belonging and connectedness to the institution. Qualitative data analysis revealed that participants were also dealing with a lack of belonging to the profession. Future work would benefit from assessing changes in professional socialization and identity through mentoring. A multi-institution study may be warranted to increase the sample size and take the model to scale. Finally, the six-month intervention is a limitation in this study and additional gains may have been made with a longer mentoring program, particularly as participants reported that virtual relationships took longer to establish.

## 5. Conclusions

A shortage of minority faculty decreases access to minority mentors for REM health sciences students, potentially contributing to inequitable educational outcomes [47,48]. This study highlights that a virtual multiple mentor model (which includes racially con-cordant peer mentoring) can decrease isolation and promote social belonging for REM health sciences students, offering an available form of support for students even after the pandemic. Students enrolled in online clinical graduate programs who express social or racial isolation may benefit from such a program. Previous research has illustrated that all members of the mentoring team make gains, not just the mentees. [18] Phase two of the study will examine the effects of the multiple-mentor model on faculty and peer mentors.

## Figures and Tables

**Table 1:** Demographics of first year graduate students, mentees, and comparison group.

Demographic Data	First Year Graduate Students (All) (N = 336)	Intervention Group (*n* = 8)	Comparison Group (*n* = 16)
Program			
CSD	63	--	6
DPT	72	4	3
GC	20	--	1
NS	101	4	4
OTD	36	--	1
PA	44	--	1
Ethnicity			
Asian	44 (13.1%)	2 (25%)	2 (12.5%)
Black/African American	23 (6.8%)	4 (50%)	--
Hispanic	24 (7.1%)	2 (25%)	--
White	223 (66.4%)	--	13 (81.3%)
Mixed Race	17 (5.1%)	--	1 (6.3%)
Unknown	5 (1.5%)	--	--

**Table 2:** Mentoring survey findings at mid-point and end of study for intervention and comparison groups.

Critical Elements and Number of Participants Who Agreed That Their Faculty Advisor Reviewed the Following Areas during Meetings	Intervention Group (*n* = 8)		Comparison Group (*n* = 15)	
	Mid-Study	End of Study	Mid-Study	End of Study
Coursework	7 (87.5%)	7 (87.5%)	9 (56.3%)	12 (75%)
Clinical education experiences	5 (62.5%)	7 (87.5%)	6 (37.5%)	8 (50%)
Professional development	6 (75%)	4 (50%)	4 (25%)	7 (43.8%)
My challenges thus far in the program	7 (87.5%)	8 (100%)	11 (68.8%)	11 (68.8%)
My successes thus far in the program	4 (50%)	6 (75%)	7 (43.8%)	10 (62.5%)
My concerns	7 (87.5%)	8(100%)	12 (75%)	10 (62.5%)

**Table 3:** Average pre- and post-College Experience Questionnaire (CEQ) scores for intervention and comparison groups.

College Experience Questionnaire (CEQ)	Intervention Group Mean (SD)		Comparison Group Mean (SD)	
	Mid-Study	End of Study	Mid-Study	End of Study
Total score	70.25 (7.62)	66.00 (11.11)	72.73 (8.52)	70.80 (4.96)
Subscales:				
University connectedness	32.38 (4.93)	30.75 (4.77)	33.4 (4.67)	31.67 (4.85)
University environment	29.38 (5.32)	27.13 (7.75)	33.00 (4.38)	32.47 (3.11)
University alienation	8.50 (3.21)	8.13 (2.85)	6.33 (1.54)	6.67 (2.32)
Sample individual CEQ items:				
I feel fully entitled to all of the resources available on campus	3.6	3.9	2.8	3.1
I feel socially alienated at this institution	2.6	2.3	2.4	2.4
I feel racially isolated	3.0	2.7	1.6	1.5
I believe that there are enough resources on campus to deal with any racial or cultural issue a student may have	3.1	3.3	3.6	3.1
There are sufficient minority faculty and staff to serve as resources for students	2.5	2.4	2.8	2.6

Note. Individual items scored on a Likert scale 1–5 (1 = strongly disagree, 5 = strongly agree).

## Data Availability

Not applicable.

## Appendix A. Mentee Interview Protocol

1. What has your experience at this institution been like? How has the intervention shaped feelings of connectedness or alienation within the university community?
2. Did this mentoring program enhance your educational and social experience as a first-year graduate student? If so, how?
3. In what ways do you feel that the mentoring program facilitates first-year graduate students’ successful transition to graduate school?
4. What is the greatest strength of this program?
5. What has been your most positive experience in the mentoring program?
6. What was your favorite program activity provided by the mentorship program?
7. What components of the program were more effective than others?
8. What are the challenges of being in a mentoring relationship?
9. What suggestions do you have to make the mentorship program more effective and beneficial for faculty, peer mentors, and mentees?
10. Is there anything else that you would like to mention about your experience in the mentorship program that I haven’t already covered?
